# Double-Blind, Placebo-Controlled, Dose-Escalating Study Evaluating the Safety and Immunogenicity of an Epitope-Specific Chemically Defined Nanoparticle RSV Vaccine

**DOI:** 10.3390/vaccines11020367

**Published:** 2023-02-06

**Authors:** Isabel Leroux-Roels, Jacques Bruhwyler, Lilli Stergiou, Mark Sumeray, Jasper Joye, Cathy Maes, Paul-Henri Lambert, Geert Leroux-Roels

**Affiliations:** 1 Center for Vaccinology (CEVAC), Ghent University Hospital, Corneel Heymanslaan 10, B-9000 Ghent, Belgium; 2 Expert Clinical Services Organization (ECSOR) sa/nv, Rue de la Station 78, B-1630 Linkebeek, Belgium; 3 Virometix AG, Wagistrasse 14, 8952 Schlieren, Switzerland; 4 Department of Paediatrics, Gynecology and Obstetrics, University of Geneva, Rue du Général Dufour 24, 1211 Geneva, Switzerland

**Keywords:** respiratory syncytial virus, RSV, vaccine, phase I, healthy volunteers, safety, immunogenicity, intramuscular, FsIIm epitope

## Abstract

**Simple Summary:**

V-306 is a synthetic virus-like particle-based vaccine candidate displaying multiple respiratory syncytial virus (RSV) F site II protein mimetics (FsIIm) as an antigenic epitope. This first-in-human, double-blind, placebo-controlled, dose-escalating study in healthy young women showed that it was safe and induced an increase in immunoglobulin G specific of FsIIm. This did not translate into an increase in RSV-neutralizing antibody titers, which were already high at baseline.

**Abstract:**

Background: V-306 is a virus-like particle-based vaccine candidate displaying respiratory syncytial virus (RSV) F site II protein mimetics (FsIIm) as an antigenic epitope. Methods: This was a randomized, placebo-controlled, double-blind, dose-escalating, first-in-human study, conducted in 60 women aged 18–45 years. Twenty subjects per cohort (15 vaccine and five placebo) received two V-306 intramuscular administrations on Days 0 and 56 at 15 µg, 50 µg, or 150 µg. Safety and immunogenicity were assessed after each vaccination and for 1 year in total. Results: V-306 was safe and well tolerated at all dose levels, with no increase in reactogenicity and unsolicited adverse events between the first and second administrations. At 50 µg and 150 µg, V-306 induced an increase in FsIIm-specific immunoglobulin G (IgG) titers, which lasted at least 4 months. This did not translate into an increase in RSV-neutralizing antibody titers, which were already high at baseline. No increase in the anti-F protein-specific IgG titers was observed, which were also high in most subjects at baseline due to past natural infections. Conclusions: V-306 was safe and well-tolerated. Future modifications of the vaccine and assay conditions will likely improve the results of vaccination.

## 1. Introduction

Respiratory syncytial virus (RSV) infections cause a substantial disease burden in the infant, immunocompromised, and elderly populations. It is estimated to induce between 55,000 and 200,000 deaths in children under 5 years of age annually, and it is associated with approximately 177,000 hospitalizations and 14,000 deaths annually in US adults aged 65 years or older [1,2,3].

Guidelines recommend the use of palivizumab (PVZ) in infants with bronchopulmonary dysplasia and in children with congenital heart disease or with severe combined immunodeficiency [4,5]. No licensed vaccine is currently available for pregnant women and children, and vaccine-associated enhanced respiratory disease (VAERD) has dramatically complicated and delayed the development of candidate RSV vaccines [1,6].

Four important proteins of the RSV virion could constitute vaccine targets: the attachment glycoprotein (G), the fusion (F) protein, the matrix (M) protein, and the small hydrophobic (SH) protein. The predominant neutralizing antibodies observed in humans are directed against the F protein (RSV F), which enables the virus to fuse with the membrane of respiratory cells [1]. Maternal vaccination from at least 3 months prior to delivery is one strategy to protect infants against RSV via transplacental transfer of maternal antibodies, while avoiding VAERD [6,7].

Virometix (Schlieren, Switzerland) developed V-306, a synthetic virus-like particle (SVLP)-based vaccine candidate displaying multiple RSV F site II (FsII, PVZ binding site) protein mimetics as the antigenic epitope. The V-306 vaccine isolates a key neutralizing epitope to ‘instruct’ the immune system to respond effectively and in a targeted manner, omitting epitopes that might mask the overall protective response. V-306 is derived from the synthesis of a conformationally constrained synthetic B-cell peptide epitope (mimetic) and its conjugation to synthetic nanoparticles made from self-assembling lipopeptides that contain a T-helper epitope and a toll-like receptor (TLR) ligand. For the design of the epitope mimetic, the crystal structure of the Fab fragment of motavizumab (an affinity-matured variant of PVZ) in complex with a peptide encompassing the RSV F glycoprotein residues Asn254–Asn277 was used, and further stabilizing sequence modifications and disulfide bridges were added [8]. The solution structure of this peptide (FsIIm) superimposed very closely on that of the motavizumab-bound peptide, proof of being a structural mimetic of the antigenic epitope on RSV site II. The nanoparticle lipopeptide backbone contains a universal CD4^+^ T helper epitope, a coiled-coil motif that forms a stable helical trimer, and the TLR-2/6 ligand Pam2Cys at the N-terminus. The FsIIm peptide was chemically linked to this lipopeptide and, in a buffered solution, gave rise to the V-306 nanoparticle-based vaccine candidate. Analysis with dynamic light scattering and transmission electron microscopy revealed particles with a mean hydrodynamic radius (Rh) of ca. 13 nm and a polydispersity index of 0.05, consistent with the formation of highly monodisperse nanoparticles of about 25–30 nm diameter. Computer modeling and prior work showed that V-306 should present the antigenic epitope in 60–90 copies exposed on its surface, whereas the lipid chains were buried in the core of the particle [9,10]. Synthetic nanoparticles designed with optimal surface properties and an optimal size range demonstrated efficient dendritic cell-mediated delivery of folded B-cell and linear T-cell epitopes into lymphoid tissues, along with ligands for pattern recognition receptors. At the same time, the multivalent display of the epitope mimetics on the surface of the nanoparticle is suitable for crosslinking B-cell receptors. In this highly immunogenic format, with the incorporation of key elements required for activation of the innate and adaptive immune systems, strong epitope-specific humoral immune responses can be elicited that target infections caused by pathogens [11,12]. Preclinical studies in mice demonstrated that V-306 vaccination leads to a significant increase in FsII-specific antibodies. These antibodies are capable of competing with PVZ for its target antigen and of neutralizing RSV. V-306 significantly decreased lung viral replication in mice after intranasal RSV challenge, without inducing enhanced RSV disease. Moreover, passive transfer of anti-V-306 isolated monoclonal antibodies protected cotton rats against viral replication in the lungs with a fourfold higher efficacy than PVZ [8,13].

The present study aimed to assess the safety and immunogenicity of V-306, administered for the first time to healthy young adult women.

## 2. Methods

This was a randomized, placebo-controlled, double-blind, sequential, parallel-cohort, dose-escalation phase 1 study conducted at the Center for Vaccinology (Ghent University and Ghent University Hospital), in accordance with Good Clinical Practice. The study was approved by the Ethics Committee of the Ghent University Hospital (Ghent, Belgium) and by the Belgian Health Authorities (Federal Agency for Medicines and Health Products). Written informed consent was obtained from all participants. An independent Data Safety Monitoring Board (DSMB) regularly reviewed the data and advised the sponsor regarding dose escalation. The EudraCT number was 2018-003277-10, and the ClinicalTrials.gov number was NCT04519073.

A total of 60 healthy women, aged 18–45 years, were included in the study. The exhaustive list of eligibility criteria, as well as subject elimination criteria from the per protocol (PP) immunogenicity cohort, can be found in the Supplementary Methods. The main exclusion criteria were a history of respiratory disease or active respiratory disease (chronic obstructive pulmonary disease, asthma, asthmatic bronchitis, dyspnea, wheezing, severe allergy) requiring concomitant medication/treatment, a past or active autoimmune disease, active smoking (more than 10 cigarettes/day), pregnancy or plan to become pregnant, and exposure to young children.

**Figure 1:**
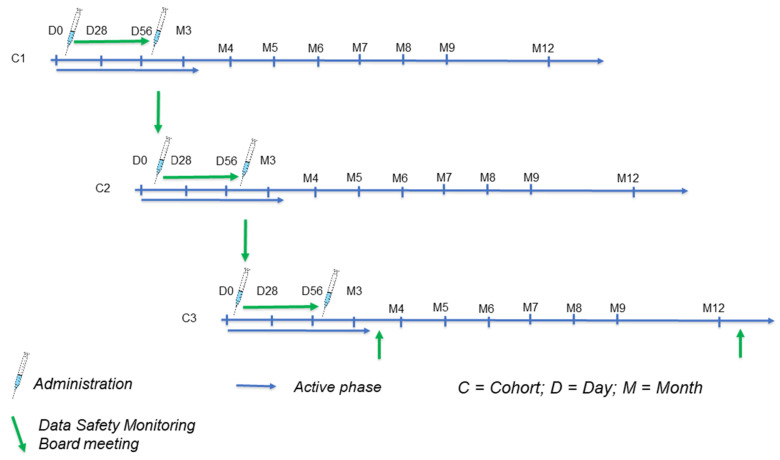
Clinical study design.

Three cohorts (N = 20 subjects per cohort) were randomized sequentially, according to a 1:3 ratio between placebo (n = 5) and a low dosage (15 µg/administration, n = 15), intermediate dosage (50 µg/administration, n = 15), or high dosage (150 µg/administration, n = 15) of V-306, administered twice according to a Month 0–Month 2 schedule (administrations on Day 0 and Day 56) (Figure 1). In each cohort, four sentinel subjects (two placebo and two vaccine subjects) were enrolled. If no holding or stopping rules applied 7 days after the administration of the test article in sentinel subjects, the enrolment was extended to the rest of the cohort (N = 16). After each administration, the subject was observed for 2 h in the investigational center, before being discharged. Exhaustive details concerning randomization and blinding processes can be found in the Supplementary Methods.

An active treatment phase from Day 0 to Month 3, i.e., 1 month after the second administration, was followed by a long-term safety follow-up phase from Month 3 to Month 12. Safety and reactogenicity were the primary endpoints of the study, while immunogenicity was a secondary or exploratory endpoint. During the active phase, subjects completed diary cards for 7 days after each administration to record solicited local signs (pain, induration, erythema, and swelling) and systemic symptoms (headache, fatigue, generalized myalgia, and fever). Other non-solicited adverse events (AEs) were recorded for 28 days after each administration. Adverse events of special interest (AESI; e.g., VAERD, lower-respiratory-tract infections, dyspnea, wheezing, cough, asthma, and influenza, RSV or SARS-CoV-2 infections), and serious adverse events (SAEs) were recorded throughout the study. Subjects visited the clinical site for safety monitoring on Days 1, 7, and 28 following each administration. Laboratory safety parameters (biochemistry, hematology, and urinalysis) were determined at screening as well as on Days 0, 1, 7, 28, 56, 57, 63, and 84. During the follow-up phase, visits for safety monitoring were scheduled at Months 6, 9, and 12 after the first administration. Humoral immunity was measured in serum on Days 0, 28, 56, and 84, as well as at Month 6, Month 9, and Month 12. The immunoassays are described in the Supplementary Methods.

The software SPSS (Version 27.0) was used throughout the statistical analyses. Descriptive statistics were used to characterize the enrolled population at baseline, as well as all the safety variables. For continuous variables, descriptive statistics included the mean, standard deviation (SD), median, minimum, and maximum. For discrete variables, descriptive statistics consisted of numbers, percentages, and cumulative percentages. Descriptive statistics were also used to characterize the immunogenicity variables in each cohort of 15 evaluable subjects receiving the candidate vaccine and in the three pooled groups of placebo at each timepoint. ELISA concentrations and neutralization titers were first transformed using a logarithm (base 2) and a logarithm (base 10) calculation, to derive the geometric mean concentration (GMC) and the geometric mean titer (GMT). Fold increases in ELISA and neutralization titers/concentrations were calculated using the log_2_-transformed values. The following exploratory statistical analyses were performed: the immunological results were compared between cohorts using Kruskal–Wallis tests, followed by post hoc two-by-two Mann–Whitney tests when significant (*p* < 0.05). Within each cohort, the immunogenicity parameters measured at the different timepoints were compared to baseline levels using Friedman’s tests, followed by post hoc two-by-two Wilcoxon tests when significant (*p* < 0.05). Details concerning the sample size and power calculation can be found in the Supplementary Methods.

## 3. Results

### Study Population Demographics and Baseline Characteristics

**Figure 2:**
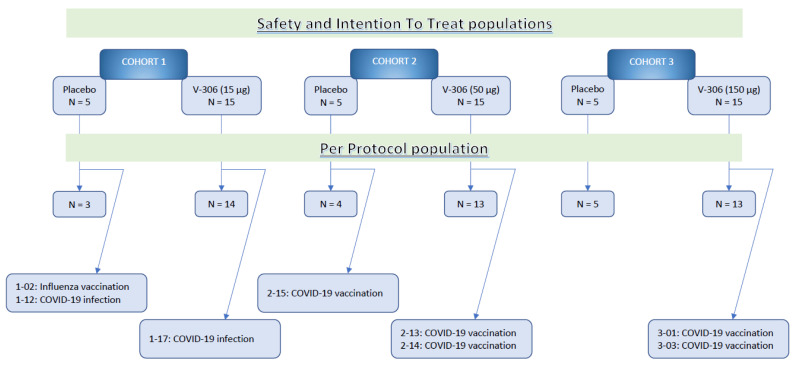
Study CONSORT diagram.

A total of 60 subjects were enrolled into three cohorts of 20 subjects (five placebo subjects and 15 vaccine subjects receiving 15 µg, 50 µg, or 150 µg of V-306) between 7 September 2020 and 9 March 2021. Those 60 subjects constituted the safety population (SP) and the intention-to-treat (ITT) population. A total of eight subjects were eliminated from the PP cohort, either for intercurrent COVID-19 infection or for vaccination (Figure 2). All subjects received two injections of the placebo/vaccine on Day 0 and Day 56, and all completed the study (last subject last visit, 2 March 2022).

The 60 female subjects (58 Caucasians, one Black, and one White/Nigerian) were 28.5 ± 8.6 years old (mean ± SD). Their BMI was 23.6 ± 4.0 kg/m². Demographic characteristics of the three vaccine cohorts and placebo pooled cohorts were similar (Supplementary Table S1).

## 4. Reactogenicity and Safety

Mild to moderate injection site pain was the most frequently reported local symptom by the majority of subjects with the three vaccine doses. There were no severe (grade 3) local symptoms. Mild to moderate headache and fatigue were the most frequently systemic symptoms reported by five subjects in the placebo group (33.3%), and by seven (46.7%) to nine (60.0%) subjects in the three vaccine groups. A case of severe (grade 3) fatigue was reported by one vaccine subject in the 15 µg group (6.7%) (Table 1). The solicited symptoms lasted 1–3 days on average.

Unsolicited AEs were reported by 10 placebo recipients (66.7%), 13 subjects in the V-306 15 µg group (86.7%), 10 subjects in the V-306 50 µg group (66.7%), and nine subjects in the V-306 150 µg group (60.0%) (Supplementary Table S2). There was only one severe (grade 3) unsolicited AE (abdominal pain) reported in one placebo subject (6.7%), which was not considered related to the investigational product by the INVESTIGATOR. During the active phase of the study, acute respiratory infections and influenza-like illness episodes were reported in three placebo recipients (20.0%), seven subjects in the V-306 15 µg group (46.7%), one subject in the V-306 50 µg group (6.7%), and three subjects in the V-306 150 µg group (20.0%). SARS-CoV-2 infection was confirmed in one placebo recipient (6.7%) and one subject in the V-306 15 µg group (6.7%). RSV and influenza were negative in all subjects. The following 14 AESIs (all unrelated to the vaccine/placebo) were reported during the long-term safety follow-up phase: SARS-CoV-2 infection (N = 6), sinusitis (N = 1), respiratory-tract infection (N = 3), upper-respiratory-tract infection (N = 2), metapneumovirus infection (N = 1), and nasopharyngitis (N = 1). No SAEs were reported during the active phase of the study. Four SAEs were reported by three subjects during the long-term follow-up phase of the study: intervertebral disc protrusion, ligament rupture, and appendicitis with incisional hernia. None of them was considered related to the candidate vaccine. Safety laboratory analyses did not reveal any clinically significant abnormalities.

## 5. Humoral Immune Response

**Figure 3:**
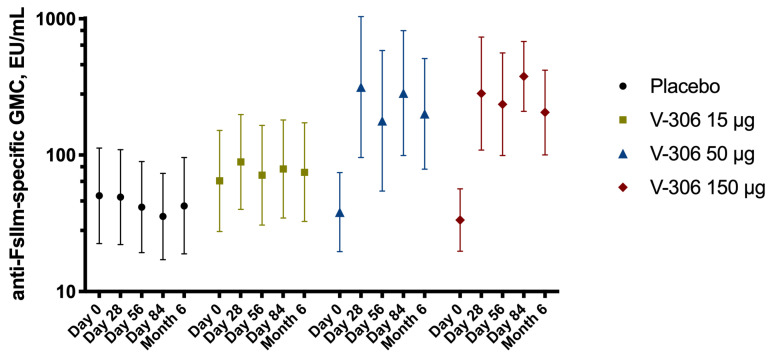
Evolution over time of anti-RSV F protein antigenic site II (FsIIm)-specific immunoglobulin G geometric mean concentrations (GMCs ± 95% confidence interval [CI]) from baseline (Day 0 pre-vaccination) up to Month 6 (4 months after second administration) in the pooled placebo and three V-306 vaccine groups (15 µg, 50 µg, and 150 µg).

The V-306 candidate vaccine induced a statistically significant increase as compared to pre-vaccination levels (*p* = 0.016 on Day 28; *p* = 0.028 on Day 56; *p* = 0.002 on Day 84; *p* = 0.026 on Month 6) in humoral responses, in terms of anti-FsIIm epitope-specific IgG. In the V-306 50 µg and 150 µg groups, there was an increase in GMCs between Day 0 and Day 28, followed by a slight decrease between Day 28 and Day 56. The second administration slightly increased the GMCs on Day 84 but without a substantial boost effect. No such increase in the IgG levels was observed in the 15 µg or placebo groups. GMCs values in the 50 µg and 150 µg groups remained high until Month 6 (4 months after second administration) with levels similar to Day 56 levels (values measured before administration of second dose), attesting to the persistence of the observed immune response (Figure 3).

High baseline values (Day 0) of microneutralization titers of RSV-A (GMT varying between 608 and 1036) and RSV-B (GMT varying between 866 and 1455) were observed in all subjects, presumably due to the fact that they were all primed by natural infection. No prescreening for anti-RSV serostatus was performed to exclude participants with high pre-existing antibody titers. No significant changes in anti-RSV-A microneutralization titers were measured between Day 0 and post-vaccination timepoints in the placebo group, as well as in the three vaccine groups. Increases in RSV-B neutralization titers were observed in one, two, and three subjects of the 15 µg, 50 µg, and 150 µg dose cohorts, respectively, at some of the timepoints tested (data not shown). Two placebo recipients also showed a rise (at least twofold) in RSV-B neutralization titers. In one of them, this could have been due to a subclinical RSV infection, not reported as an unsolicited AE.

Baseline levels of anti-F protein-specific IgG were also high in all participants, and no significant changes were observed following administration of V-306 or placebo (data not shown).

**Figure 4:**
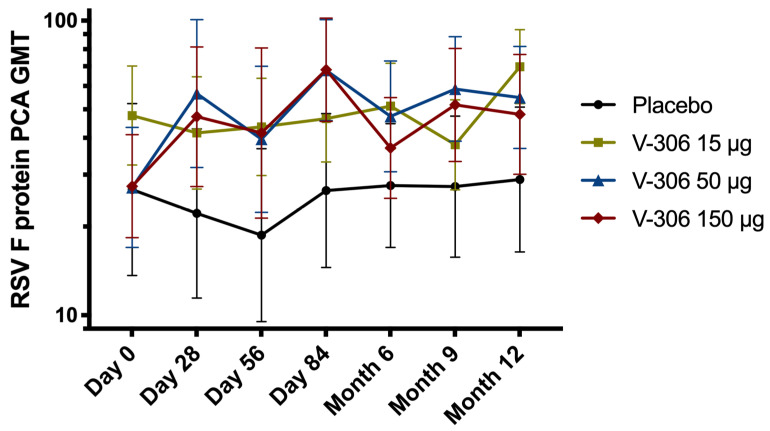
Evolution over time of serum antibodies geometric mean titers (GMTs ± 95% confidence interval [CI]) competing with palivizumab for binding to the RSV F protein antigenic site II (FsIIm) from baseline (Day 0 pre-vaccination) up to Month 12 (10 months after second administration) in the pooled placebo and three V-306 vaccine groups (15 µg, 50 µg, and 150 µg).

A PVZ-competing antibody (PCA) assay was used to quantify the levels of serum antibodies that compete with PVZ for binding to the RSV F protein antigenic site II, as a means to assess the PVZ-like antibody responses triggered by the vaccine. There was a statistically significant increase in PCA titers in the 50 µg (*p* = 0.008) and 150 µg (*p* = 0.004) vaccine groups compared to placebo on Day 84 (1 month after second administration) with persistence until Month 9 (Figure 4).

## 6. Discussion

Over the years, approaches with traditional vaccinology have faced difficulties in delivering effective vaccines. Whole inactivated virus candidates cause VAERD and unfocused antibody responses with reduced neutralization [14], and post-fusion forms of RSV F similarly produce less potent neutralizing antibodies [15]. Stabilized forms of the pre-fusion RSV F do exhibit more epitopes than post-fusion forms [16], and breathable, prefusogenic forms of RSV F provide accessibility to antigenic epitopes including intermediate conformations [17]. Moreover, mRNA vaccines carrying the information of the entire pre-fusion F protein have demonstrated very good efficacy in man [18,19]. As opposed to antigens coding for the entire RSV F protein, Virometix has taken an epitope-focused approach [20], on a target that is found on both the pre- and the post-fusion forms of RSV F. This approach uses a key neutralizing epitope to ‘guide’ the immune system to respond in a targeted manner, omitting epitopes where raised antibodies might mask the protective response. Specifically, the V-306 vaccine targets the only clinically approved mAb-binding site, trying to provide similar and/or superior to PVZ protective antibodies through a vaccination approach.

This first-in-human clinical study shows that V-306 is a safe and well-tolerated RSV vaccine candidate. No clear dose–effect relationship was found in terms of reactogenicity and safety. The second administration was not more reactogenic than the first. The maximum tolerated dose was not reached. No SAEs considered related to the vaccine were reported and no VAERD was detected.

V-306 (medium [50 µg] and high doses [150 µg]) induced a statistically significant increase in humoral responses, in terms of anti-FsIIm epitope-specific IgG, from 28 days after injection. The second dose did not substantially increase antibody levels, although a small increase was observed. GMCs remained high until Month 6 (4 months after second administration), demonstrating the persistence of the observed immune response. The presence and magnitude of increase in anti-FsIIm epitope-specific IgG is consistent with the observed increase in PVZ competing activity in serum of participants. V-306 did not induce an increase in the RSV-A neutralization titers that were already very high at baseline, and only a few subjects showed a rise in RSV-B neutralization titers. Baseline (pre-vaccination) levels of anti-F protein-specific IgG were high in most subjects as a consequence of past natural infections. Administration of V-306 did not lead to an increase in the anti-F protein baseline levels, as no differences between vaccinated subjects and the placebo group were observed.

The gold standard for measuring functional antibody responses following vaccination with RSV vaccines is the neutralization assay, whereas, for the demonstration of an increase in F protein-binding antibodies, ELISA assays are used. However, discordant results have been observed with these two immunoassays following vaccination with post-fusion F protein antigens. Some clinical trials have shown that the increase in binding antibody far exceeded the improvement in neutralizing activity [15,21], indicating that post-fusion F protein vaccines, while being considered immunogenic, elicited primarily non-neutralizing or only weakly neutralizing antibodies. The antibody responses to a wide variety of epitopes displayed on the F protein can be measured with neutralization and ELISA assays, but they do not provide information on the fine specificity of these responses, with respect to the recognition of different antigenic sites or preference for pre- or post-F conformation. A multiplex assay that detects and quantifies the interaction of polyclonal serum antibodies with an array of critical neutralizing epitopes would allow a more refined analysis of the repertoire of circulating antibodies.

The efficacy of PVZ has been demonstrated in high-risk infants. On that basis, immunoassays have been developed to measure the immune responses that compete with PVZ for binding to the F protein. It seems logical to assume that antibodies that compete with PVZ should also protect from the disease and, therefore, that PCA serum concentrations could serve as surrogate for neutralization. PCA levels are, therefore, routinely conducted to measure RSV vaccine-elicited immunity. However, most of these PCA measurements have been performed with a post-fusion F protein or a structurally undefined antigen [22,23,24,25,26]. Taking into account the development of several pre-fusion F protein candidate vaccines, as well as our current knowledge of the F protein biology, it now appears that this is an inadequate and potentially misleading immunological readout [27].

New vaccine development pathways are strongly influenced by technologies that define the atomic-level structure of neutralization-sensitive epitopes on viral surface proteins. Preserving the prefusion conformation of the RSV F glycoprotein has been a challenge for protein engineers, resulting in a stabilized subunit vaccine candidate (DS-Cav1). This candidate has shown promising preclinical results in mice and monkeys. In a phase 1 clinical trial, there was more than a tenfold increase in serum neutralizing activity resulting from antibodies targeting prefusion-specific surfaces of the RSV F protein [28]. Preliminary exploratory data revealed that the V-306 study subjects displayed higher titer changes in PCA assays when the pre-fusion F protein conformation of RSV F was used as the capturing antigen (unpublished data), likely reflecting a better accessibility of the relevant epitope on the pre-fusion F protein. This may also reflect the diversity of pre-existing neutralizing and non-neutralizing antibodies [29], and their capacity to interfere with the binding of anti-FsIIm epitope-specific IgG to the PVZ-binding epitope [30].

Although V-306 induced a robust and specific increase in IgG binding to the FsIIm epitope in the two highest dose cohorts, this did not translate into the expected increase in titers of neutralizing antibodies against RSV-A and -B in vitro. The IgG response was, however, associated with an increase in titer of competing IgG against PVZ for binding to antigenic site II on the F protein. Since the population of subjects in our study demonstrated high baseline levels of neutralizing IgG titer against RSV-A and -B, it is likely that their serum contained a relatively high quantity of IgG (both neutralizing and non-neutralizing) with binding affinity for multiple epitopes on the whole F protein. The presence of this pre-existing IgG may have resulted in some degree of competition with V-306-specific IgG for access to FsIIm, as well as potential interference by steric hindrance caused by binding to closely proximate epitopes. This raises the possibility that the IgG response elicited by V-306 may not have been sufficiently robust to overcome the impact of baseline immunity. In an unpublished experiment (Supplementary Figure S1), we spiked two serum samples (one considered to be negative for PVZ competing antibodies and one considered positive with a titer of 160) with PVZ (4 to 108 µg/mL). The resulting increase in PCA titers was much higher in the negative than in the positive samples. On the basis of these results, it seems likely that, in subjects positive for PVZ-competing antibodies at baseline, an increase in PVZ-like antibodies (e.g., upon vaccination) might not be reflected in the measurement. It is, therefore, possible that the results after vaccination in these subjects might be an underestimation.

Nevertheless, the present study successfully demonstrated the acceptable safety and tolerability profile of the SVLP vaccine platform, thereby reinforcing the potential to further explore next generation candidates against RSV and other pathogens of interest. Moreover, the SVLP particle as an antigen delivering vehicle is an immunogenic platform and generates the correct type of antibodies, i.e., the intended ones (PVZ-like), according to the designed epitope mimetic.

## 7. Conclusions

In conclusion, several future modifications of the vaccine, as well as refinements to the study population and assay conditions, will likely improve the results of vaccination. One of them would be to increase the administered dose to potentially obtain higher levels of epitope-specific antibodies, such that they exceed the level of pre-existing immunity. Along the same lines, the vaccine could be tested in subjects with lower baseline immunity to assess the mere effect of the vaccine in a background free of obstructing antibodies. Lastly, toward a more effective vaccine design, the addition of one or two additional antigenic epitopes in the vaccine mimicking additional neutralizing epitopes could be a viable strategy. For example, the sites F0 and FIV on the RSV F protein are targets of monoclonal antibodies currently in development [19,31,32,33]. Such sites are starting points for generating peptide mimetics, which focus antibody responses to sites of virus vulnerability. Furthermore, standardization/optimization of assay conditions, such as performance of PCA with the use of the pre-F form of the RSV F protein might shed more light on the antibody quality.

## Figures and Tables

**Table 1:** Number and percentage of subjects reporting solicited local and systemic signs or symptoms during 7 days after the two administrations (Days 0 and 56) in the pooled placebo groups and in the vaccine groups (V-306 15 µg, 50 µg and 150 µg).

	Placebo N = 15		V-306 15µg N = 15		V-306 50µg N = 15		V-306 150µg N = 15	
	n	%	n	%	n	%	n	%
At least one local symptom	1	6.7	13	86.7	13	86.7	15	100.0
At least one local severe symptom	0	0.0	0	0.0	0	0.0	0	0.0
Pain	1	6.7	13	86.7	13	86.7	15	100.0
Induration	0	0.0	1	6.7	2	13.3	5	33.3
Erythema	0	0.0	1	6.7	0	0.0	0	0.0
Swelling	0	0.0	0	0.0	0	0.0	1	6.7
At least one systemic symptom	7	46.7	9	60.0	13	86.7	12	80.0
At least one systemic severe symptom	0	0.0	1	6.7	0	0.0	0	0.0
Headache	5	33.3	8	53.3	9	60.0	7	46.7
Fatigue	5	33.3	7	46.7	9	60.0	9	60.0
Fatigue (severe)	0	0.0	1	6.7	0	0.0	0	0.0
Generalized myalgia	2	13.3	3	20.0	0	0.0	1	6.7
Fever	0	0.0	0	0.0	0	0.0	0	0.0

## Data Availability

The data presented in this study are available on request from the corresponding author.

## Supplementary Materials

The following supporting information can be downloaded at: https://www.mdpi.com/article/10.3390/vaccines11020367/s1, Supplementary Figure S1. Modification of Palivizumab competing antibody (PCA) titers as a function of palivizumab (PVZ) concentration spiked in the serum samples of two subjects: VC-2120130009: considered to be negative for PVZ competing antibodies and VC-2120130010: considered positive with a baseline titer of 160. Supplementary Table S1. Demographics and baseline subject characteristics. Supplementary Table S2. Number and percentage of subjects reporting unsolicited adverse events during 28 days after each of the two administrations (Day 0 and Day 56), classified according to the Medical Dictionary for Regulatory Activities (MedDRA) into System Organ Class and Preferred Term, in the placebo and vaccine cohorts (Cohort 1 = V-306 15 µg; Cohort 2 = V-306 50 µg and Cohort 3 = V-306 150 µg). Ref. [34] is cited in the supplementary materials.
