# Supplementary material for: Double-Blind, Placebo-Controlled, Dose-Escalating Study Evaluating the Safety and Immunogenicity of an Epitope-Specific Chemically Defined Nanoparticle RSV Vaccine
Source: Vaccines (Basel). 2023 Feb 6;11(2):367. doi: 10.3390/vaccines11020367 (PMC9967611; doi:10.3390/vaccines11020367)
Supplement: Supplementary file 1 [file vaccines-11-00367-s001.zip › vaccines-2184111-supplementary.pdf]

SUPPLEMENTARY METHODS

Inclusion criteria

The following criteria were checked at the time of screening and Day 0 (pre-first administration). The subject could only be included in the study if all inclusion criteria were fulfilled:

- Written informed consent.
- Healthy women aged between 18-45 years.
- No evidence of disease based on medical history, physical examination, vital signs (blood pressure, heart rate, body temperature and respiratory rate), laboratory safety parameters and clinical judgement.
- Not pregnant and committed to not becoming pregnant during the whole study period. Committed to use adequate and effective contraception means in accordance with the Clinical Trial Facilitation Group [CTFG] criteria, as follows: "*Contraceptive methods that can achieve a failure rate of less than 1% per year when used consistently and correctly are considered as highly effective birth control methods. Such methods include: combined (estrogen- and progestogen-containing) hormonal contraception associated with inhibition of ovulation (oral, intravaginal, transdermal), progestogen-only hormonal contraception associated with inhibition of ovulation (oral, injectable, implantable intrauterine device, intrauterine hormone-releasing system), bilateral tubal occlusion, vasectomised partner and/or sexual abstinence*."
- The subjects must have used adequate and effective contraception means (CTFG criteria) for at least 60 days prior to the first administration.

- Capability to meet the requirements of the study.

Exclusion criteria

The following criteria were checked at the time of screening and Day 0 (pre-first administration). If any exclusion criterion applied, the subject could not be included in the study:

- Presence of serologic markers of acute or chronic Human Immunodeficiency Virus (HIV), Hepatitis B Virus (HBsAg and anti-HBc) and Hepatitis C Virus (anti-HCV) infection(s).
- As judged by the Investigator, any clinically significant disease related to the cardiovascular (CV), gastrointestinal (GI) or central nervous system (CNS).
- Any chronic disease, or history of significant disease that might interfere with the trial's conduct or completion. Some conditions may be accepted if stabilized, e.g. hypertension.
- An active respiratory disease or symptoms thereof (chronic obstructive pulmonary disease, asthma, asthmatic bronchitis, dyspnea, wheezing, severe allergy) requiring medication, or history of such disease.
- Personal history of active or past autoimmune disease
- Administration for more than three months prior of study start of immunosuppressant or immuno-modifying drugs (including systemic corticosteroids).
- Confirmed or suspected (at the discretion of the Investigator) immuno-suppressive or immuno-deficient condition.

- Current smokers (more than 10 cigarettes/day).
- Blood transfusion, blood product, immunoglobulins received during the period of 3 months prior to study start.
- Clinically significant (according to Investigator's judgement) laboratory out of range values. The abnormal lab test can be neglected if its cause is evident and of no clinical relevance
- Acute disease and/or fever (≥38°C measured by the oral route) at the time of test article administration. Vaccine administration can be postponed until the febrile episode is over.
- Recent vaccination (e.g., vaccine administration within 2 weeks or 4 weeks [live attenuated]) or evidence that a vaccine will be required during the study period (e.g., planned travel).
- Pregnant or plan to become pregnant during the study period.
- Breastfeeding.
- Women highly exposed to children less than 5 years of age will be excluded to reduce risk of RSV infection, including mothers of young children, pediatric nurses, personnel of day nursery.
- Previous participation in a RSV vaccine study.
- Any other significant finding that would increase, according to the Investigator, the risk of having an adverse outcome from participating in the study.
- History of (suspected) hypersensitivity reaction that could be triggered by any component of the vaccine.

Elimination criteria from the per protocol (PP) immunogenicity cohort

The following criteria were checked at each visit subsequent to the vaccination. If any applied during the study, it would not require withdrawal of the subject from the study but could determine a subject's evaluability in the PP analysis:

- Use of any investigational or non-registered product (drug or vaccine) other than the study product during the study period.
- Receipt of routine commercial vaccinations (with the exception of seasonal influenza vaccination) during the active phase of the study.
- Administration of more than 7 days of immune-suppressants or other immune-modifying drugs during the active phase of the study. For corticosteroids, this would mean prednisone ≥0.125 mg/kg/day (maximum 10 mg/day), or equivalent. Inhaled and topical steroids were allowed.
- Administration of immunoglobulins and/or any blood products during the active phase of the study.

Randomization and blinding processes

Within each of the 3 cohorts, the 20 subjects were randomly assigned to 2 study groups: vaccine or placebo in a 3:1 ratio (15 vaccine and 5 placebo). The block size was equal to 4. Three randomization lists (one per cohort) were prepared by the Data Management Department of the CRO ECSOR (person not involved in the study), using the software nQuery Advisor. They were communicated to the Electronic Data Capture Department of ECSOR for their integration into the eCRF platform, to the pharmacist or to the delegate

from the investigational center in charge of the preparation of syringes for injection at CEVAC and to the Independent Data Manager of the DSMB.

The study was double-blind. The Investigator, the laboratory and the subjects were kept blind to the treatment arm (placebo/vaccine) to which the subject had been allocated up to the end of the study (Month 12). Syringes containing the test article (placebo or vaccine) to be administered were prepared by an unblinded pharmacist or a delegate from the investigational center, independent of clinical staff, and were delivered to the clinical team for administration in a blinded manner. The immunogenicity data, which would lead to the unblinding of the treatment groups, were not available during the course of the study to the Investigator or any person involved in the clinical conduct of the study, until the end of the active phase (Month 3 included). The blinding at the individual subject level was maintained until the end of the follow-up phase of the study (Month 12 included). The personnel in charge of the laboratory testing was blind until the end of the study.

Sample size and power calculations

The sample size aimed to allow an initial assessment of the safety and immunogenicity of the vaccine candidate. The study was not powered for a statistical hypothesis testing. In the three pooled vaccine groups (N=36 evaluable subjects), based on the binomial distribution, there was a 70% chance (70% power) that a SAE would be detected if the true rate of occurrence of a given SAE was 3.3% and a 90% chance (90% power) if it was 6.2% (nQuery Advisor, Version 7.0). The power to detect a SAE having a true occurrence rate of 5% was 84.2% and the power to detect a SAE having a true occurrence rate of 1% was 30.4% (nQuery Advisor, Version 7.0).

ELISA for the determination of RSV anti-FsIIm serum IgG

An ELISA method was used to determine the concentration of IgG antibodies against the antigen mimetic in V-306 in human serum samples (anti-FsIIm epitope-specific IgG). Briefly, ELISA plates were coated with 2 µg/mL of the FsIIm antigen or left uncoated and without the antigen. The non-uncoated wells were used to measure non-specific interactions of each serum sample with the assay plate. Following blocking of all wells with a skimmed milk solution, test samples, standard curve samples and quality control (QC) samples were added to the ELISA plates and incubated to allow binding of antibodies to the antigen coats. After washing, HRP-conjugated anti-human IgG detection antibody (Sigma-Aldrich, A8792-2ML) was added at 1:50.000, followed by incubation and subsequent washing. The amount of antigen-specific IgG binding was subsequently quantified by measuring the absorbance after addition of TMB substrate. The absorbance of the non-coated wells was subtracted from the absorbance of the FsIIm-coated wells of each serum sample before interpolating the anti-FsIIm IgG concentration in samples as ELISA Units (EU)/mL from the standard curve by using Gen5 analysis software. A 4PL curve plot was fitted through the signal of the standard curve samples. The absorbance of each individual sample was read back to the reference curve to obtain reportable values, from which a mean reportable value was calculated based on the replicates. The antibody concentration was reported in arbitrary EU/mL, based on the assignment of a reference value in EU/mL concentration for the first dilution of the standard curve.

Palivizumab Competing Antibody (PCA) assay

The PCA assay was used to determine the titer of RSV F antigenic site II binding antibodies in serum samples. Briefly, 96-well microtiter plates were coated with RSV Fusion protein antigen (Sino Biologicals, Cat# 11049-V08B). Biotinylated Palivizumab (PVZ) was

prepared from PVZ (AbbVie, Cat# J06BB16) using the EZ-Link Sulfo-NHS-LC-Biotinylation Kit (Thermo Scientific, Cat# 21435). Unknown samples, controls (maximal and minimal signal) and a QC (Quality Control) sample were mixed with a fixed concentration of biotinylated Palivizumab (PVZ-Bio). Mixtures were subsequently added to the plates and incubated to allow binding of antibodies to the antigen coats. After washing, HRP-conjugated streptavidin (Life Technologies, Cat# N100) was added followed by incubation and subsequent washing. The amount of PVZ-Bio binding was subsequently quantified by measuring the optical density (OD) (450 and 620 nm) after addition of TMB substrate and stopping of the reaction with H2SO4. OD450-620 values were normalized against the maximum binding signal and the percentage PVZ-Bio binding at each sample dilution was calculated. Using 4-parameter curve fit analysis, the titer for each serum sample was determined as the sample dilution that resulted in 50% inhibition of PVZ-Bio binding to RSV F according to the method described by Smith et al. (2012) and Widjaja et al. (2016).

References

Smith G, Raghunandan R, Wu Y, et al. Respiratory syncytial virus fusion glycoprotein expressed in insect cells form protein nanoparticles that induce protective immunity in cotton rats. PLoS One. 2012;7(11): e50852.

Widjaja I, Wicht O, Luytjes W, et al. Characterization of Epitope-Specific Anti-Respiratory Syncytial Virus (Anti-RSV) Antibody Responses after Natural Infection and after Vaccination with Formalin-Inactivated RSV. J Virol. 2016;90(13): 5965-5977.

SUPPLEMENTARY FIGURE LEGENDS

Supplementary Figure S1. Modification of Palivizumab competing antibody (PCA) titers as a function of palivizumab (PVZ) concentration spiked in the serum samples of two subjects: VC-2120130009: considered to be negative for PVZ competing antibodies and VC-2120130010: considered positive with a baseline titer of 160.

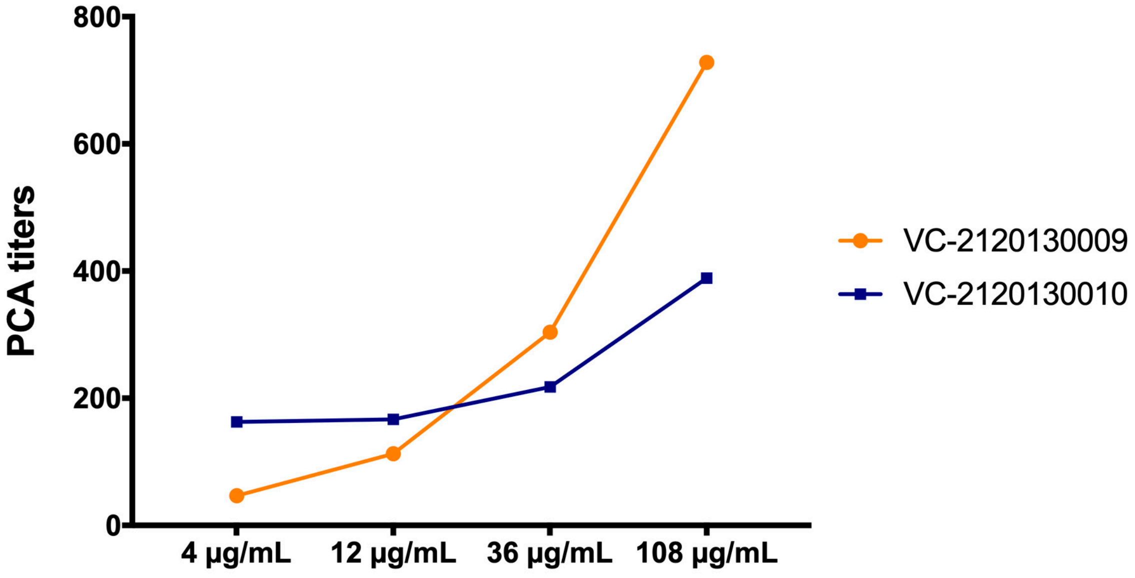

SUPPLEMENTARY TABLES

Supplementary Table S1. Demographics and baseline subject characteristics

		**V-306 15µg (N=15)**	**V-306 50µg (N=15)**	**V-306 150µg (N=15)**	**Pooled placebo (N=15)**	**All (N=60)**
Age (years)	Mean (SD)	28.1 (7.8)	30.4 (9.5)	25.3 (7.5)	30.2 (9.2)	28.5 (8.6)
	Median	25.0	28.0	21.0	27.0	25.5
	Min ; Max	19 ; 43	20 ; 45	19 ; 43	20 ; 44	19 ; 45
Body Mass Index (kg/m^2)	Mean (SD)	22.4 (3.5)	26.4 (4.4)	23.2 (3.1)	22.5 (3.9)	23.6 (4.0)
	Median	22.4	25.2	23.7	22.2	23.2
	Min ; Max	17.3 ; 32.0	22.4 ; 37.3	18.4 ; 30.5	17.3 ; 33.2	17.3 ; 37.3
Ethnicity	Caucasian, n (%)	15 (100.0)	14 (93.3)	14 (93.3)	15 (100.0)	58 (96.7)
	Black, n (%)	0 (0.0)	1 (6.7)	0 (0.0)	0 (0.0)	1 (1.7)
	Other*, n (%)	0 (0.0)	0 (0.0)	1 (6.7)	0 (0.0)	1 (1.7)
Medical history	Presence, n (%)	8 (53.3)	8 (53.3)	9 (60.0)	12 (80.0)	37 (61.7)

** Mother is White Caucasian and father is Nigerian*

Supplementary Table S2. Number and percentage of subjects reporting unsolicited adverse events during 28 days after each of the two administrations (Day 0 and Day 56), classified according to the Medical Dictionary for Regulatory Activities (MedDRA) into System Organ Class and Preferred Term, in the placebo and vaccine cohorts (Cohort 1 = V-306 15 µg; Cohort 2 = V-306 50 µg and Cohort 3 = V-306 150 µg)

**MedDRA System Organ Class**	**MedDRA Preferred term**	**Vaccine**			**Placebo**		
		**N**	**n**	**%**	**N**	**n**	**%**
		**Cohort 1**					
Blood and lymphatic system disorders	Lymphadenopathy	15	1	6.7	5	1	20.0
Gastrointestinal disorders	Nausea	15	1	6.7	5	0	0.0
	Abdominal discomfort	15	1	6.7	5	0	0.0
General disorders and administration site conditions	Injection site pain	15	1	6.7	5	0	0.0
	Fatigue	15	1	6.7	5	0	0.0
	Injection site movement impairment	15	1	6.7	5	0	0.0
	Injection site haemorrhage	15	2	13.3	5	0	0.0
Infections and infestations	Vulvovaginal candidiasis	15	1	6.7	5	0	0.0
	Eye infection	15	0	0.0	5	1	20.0
	Upper respiratory tract infection	15	0	0.0	5	1	20.0
	COVID-19	15	1	6.7	5	0	0.0
Musculoskeletal and connective tissue disorders	Neck pain	15	1	6.7	5	0	0.0
	Limb discomfort	15	1	6.7	5	0	0.0
	Myalgia	15	1	6.7	5	0	0.0
Psychiatric disorders	Depression	15	1	6.7	5	0	0.0
Respiratory. thoracic and mediastinal disorders	Oropharyngeal pain	15	3	20.0	5	1	20.0
	Rhinorrhoea	15	2	13.3	5	0	0.0
	Nasal congestion	15	3	20.0	5	0	0.0
	Productive cough	15	1	6.7	5	0	0.0
Nervous system disorder	Headache	15	0	0.0	5	1	20.0
		**Cohort 2**					
Gastrointestinal disorders	Diarrhoea	15	1	6.7	5	0	0.0
	Abdominal pain	15	0	0.0	5	2	40.0
	Toothache	15	0	0.0	5	1	20.0
	Vomiting	15	1	6.7	5	0	0.0
General disorders and administration site conditions	Discomfort	15	1	6.7	5	0	0.0
	Fatigue	15	0	0.0	5	1	20.0
	Injection site movement impairment	15	2	13.3	5	0	0.0
	Malaise	15	1	6.7	5	0	0.0
	Medical device site dermatitis	15	1	6.7	5	0	0.0
	Injection site haemorrhage	15	1	6.7	5	1	20.0
Infections and infestations	Respiratory tract infection	15	1	6.7	5	0	0.0
	Cystitis	15	1	6.7	5	0	0.0
Metabolism and nutrition disorders	Polydipsia	15	1	6.7	5	0	0.0
Musculoskeletal and connective tissue disorders	Muscular weakness	15	1	6.7	5	0	0.0
	Medial tibial stress syndrome	15	1	6.7	5	0	0.0
Nervous system disorders	Headache	15	2	13.3	5	0	0.0
	Paraesthesia	15	0	0.0	5	1	20.0
Respiratory. thoracic and mediastinal disorders	Oropharyngeal pain	15	2	13.3	5	0	0.0
Renal and urinary disorders	Dysuria	15	0	0.0	5	1	20.0

		Cohort 3					
Blood and lymphatic system disorders	Lymphadenopathy	15	1	6.7	5	0	0.0
Gastrointestinal disorders	Nausea	15	1	6.7	5	0	0.0
	Abdominal pain	15	1	6.7	5	0	0.0
General disorders and administration site conditions	Malaise	15	1	6.7	5	0	0.0
Infections and infestations	Respiratory tract infection	15	1	6.7	5	0	0.0
	Pharyngitis	15	1	6.7	5	0	0.0
	Nasopharyngitis	15	1	6.7	5	0	0.0
	Rhinitis	15	1	6.7	5	0	0.0
Nervous system disorders	Dizziness	15	1	6.7	5	0	0.0
Respiratory. thoracic and mediastinal disorders	Oropharyngeal pain	15	1	6.7	5	0	0.0
	Throat irritation	15	1	6.7	5	0	0.0
Injury. poisoning and procedural complications	Procedural pain	15	1	6.7	5	0	0.0
